# Case Report: A Case of Glioblastoma in a Patient With Haberland Syndrome

**DOI:** 10.3389/fped.2021.648717

**Published:** 2021-03-08

**Authors:** Silvia Ferranti, Iacopo Sardi, Milena Guidi, Chiara Lembo, Salvatore Grosso

**Affiliations:** ^1^Department of Molecular Medicine and Development, University of Siena, Siena, Italy; ^2^Department of Pediatric Oncology, Meyer Children's Hospital, Florence, Italy

**Keywords:** Haberland syndrome, encephalocraniocutaneous lipomatosis, glioblastoma, tumor, epilepsy

## Abstract

Haberland syndrome or encephalocraniocutaneous lipomatosis is a rare ectomesodermal dysgenesis defined by the triad including ocular, skin, and central nervous system involvement, which is commonly unilateral. This disorder is attributed to a post-zygotic mutation responsible for a neural tube and neural crest dysgenesis. We report the case of a 15-year-old female with Haberland syndrome with pharmacoresistant epilepsy who developed a World Health Organization-grade IV glioblastoma. This is the first case of pediatric glioblastoma associated with Haberland syndrome. The previously reported pediatric cases included benign brain tumors. To our knowledge, this is the fifth case of brain tumor associated with encephalocraniocutaneous lipomatosis and the second case of glioblastoma associated with this syndrome. The hypothesis that Haberland syndrome is associated with an increased risk of tumor development is intriguing, although the rarity of the condition is nowadays preventing us from drawing definitive conclusions about this potential link between the two entities. Further studies are needed to establish the real relationship between encephalocraniocutaneous lipomatosis and the risk of brain tumors.

## Introduction

Haberland syndrome or encephalocraniocutaneous lipomatosis is a rare ectomesodermal dysgenesis defined by the triad including ocular, skin, and central nervous system involvement, which is commonly unilateral (1). This disorder is attributed to a post-zygotic mutation responsible for a neural tube and neural crest dysgenesis (2). We report the case of an adolescent with Haberland syndrome who developed a glioblastoma. It is possible to hypothesize that Haberland syndrome, similarly to most of the neurocutaneous syndromes, is associated with a predisposition to cancer development.

## Case Description

We describe the case of a 15-year-old female who was diagnosed with Haberland syndrome during infancy due to the presence of clinical signs including palpebral telangectasias, facial lipomas, angiomas of the trunk, scalp alopecia, scleral dermatolipomas, irideal abnormalities, and unilateral mandibular swelling. She also presented with severe mental and speech retardation and pharmacoresistant epilepsy. At the age of 18 months, she started to present with focal motor onset seizures with automatisms and impaired awareness. During early childhood, she presented with episodes of facial pallor, perioral cyanosis, and chewing automatisms without consciousness impairment. During school-age, focal non-motor onset autonomic aware seizures were observed. Therapy included combinations of Valproate, Topiramate, Phenobarbital, Delorazepam, Clonazepam, and Rufinamide.

**Figure 1:**
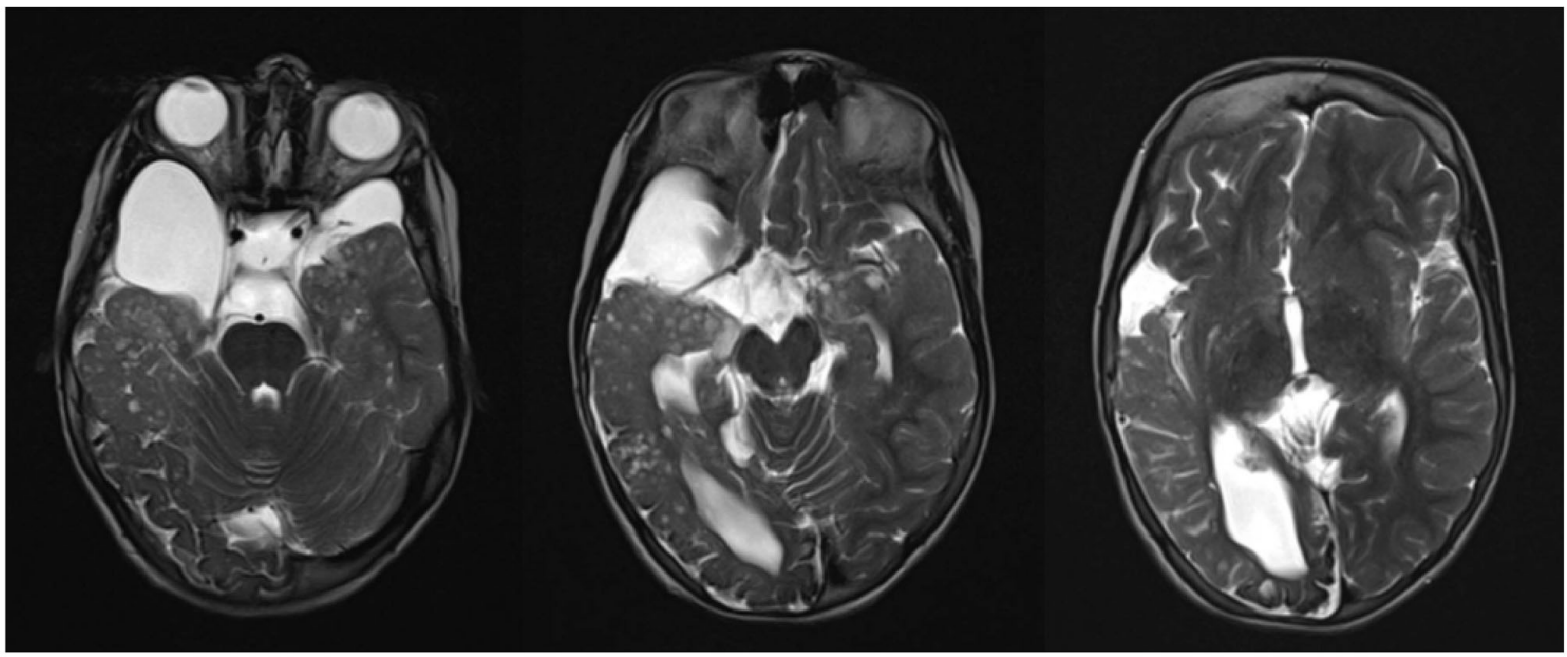
Occipital-temporal-parietal leptomeningeal angiomatosis, unilateral lipoma along the trigeminal way with adjacent arachnoid cyst, and pseudo-cystic areas of temporal lobe and thalami.

Brain magnetic resonance imaging (MRI) findings were compatible with Haberland syndrome diagnosis according to criteria of Hunter modified by Moog (2). Neuroradiological abnormalities included occipital-temporal-parietal leptomeningeal angiomatosis, unilateral lipoma along the trigeminal way with an adjacent arachnoid cyst, and pseudo-cystic areas of temporal lobe and thalami (Figure 1).

At the age of 15 the patient, who was under therapy with Valproate, Topiramate, and Rufinamide, presented with a sudden worsening of the general conditions with repeated vomiting, weight loss, and a significant increase in the frequency of epileptic seizures, which consisted of generalized onset motor tonic-clonic seizures occurring 5–6 times per day. The electroencephalogram showed background rhythm slowing in the left hemisphere and bilateral multifocal paroxysmal activity.

**Figure 2:**
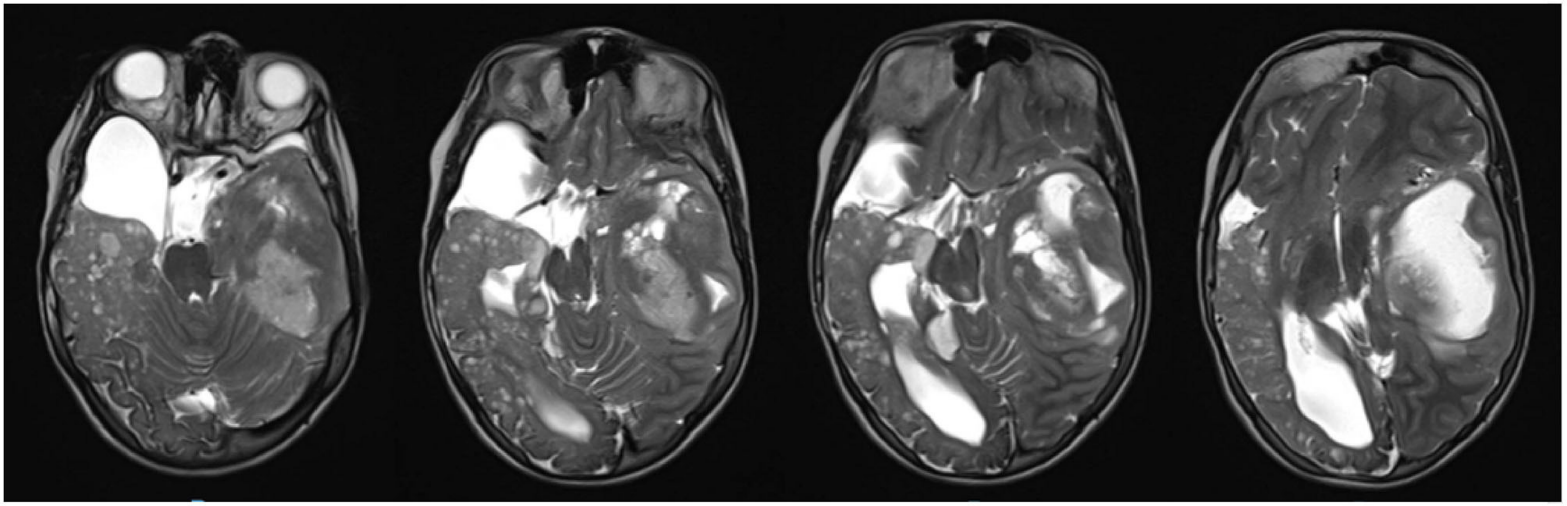
Large expansive lesion in the left temporal-insular area with a combined solid and cystic structure. Striking mass-effect on flanking structures, with right-sided midline shift, initial transtentorial herniation, and midbrain subluxation.

Brain MRI was repeated and documented a large expansive lesion in the left temporal-insular area, 7 × 4.5 × 4 cm in size, with a combined solid and cystic structure. The lesion was exerting a striking mass-effect on flanking structures, with right-sided midline shift, initial transtentorial herniation, and midbrain subluxation (Figure 2). The girl underwent gross total surgical removal of the temporal-insular lesion. Histological analysis revealed a glioblastoma (World Health Organization -grade IV) characterized by small cells with marked angiogenesis and wide necrotic areas infiltrating both cerebral structures and meningeal sheaths. Ki-67 labeling was 40–45% of all cells in the resected tumor area. The immune-histochemical profile of the neoplasm showed positivity for ATRX and GFAP whereas p53 and PDL1 were negative. Tumor cells were negative for IDH1 and IDH2 genes. The tumor was also negative for EGFR, c-Myc, and N-Myc amplification, and no MGMT promoter methylation was detected.

Histological diagnosis was examined after admission for adjuvant treatment in all cases by two pathologists. Thus, the patient underwent radiotherapy for a total dose of 59.4 Gy (1.8 Gy/fraction, five fractions per week) to the operative bed plus a 2 cm margin. The treatment was given with volumetric-modulated arc therapy. The treatment was associated with concomitant and sequential chemotherapy with Temozolomide (3). She maintained a Lansky performance score >70. Following a worsening of symptoms, she was subjected to another MRI scan that showed local recurrence. She underwent a second maximal surgery and began the Procarbazine, Lomustine, and Vincristine regimen (4).

After 10 months an MRI scan of the brain showed evidence of disease progression. Based on the complexity of her syndrome and the prognosis of relapsed glioblastoma a home supportive and palliative approach was therefore decided. Surprisingly, the girl died 11 months after the palliative care was started.

## Discussion

The most common clinical manifestations of Haberland syndrome include non-progressive mental retardation, focal epileptic seizures, hemiplegia, spasticity, and facial nerve palsy (5). Neuroradiological abnormalities include unilateral poroencephalic cysts, lipomatous hamartomas involving the scalp, eyebrows, and eyeball, arachnoid cysts, leptomeningeal granulomatosis, intracranial calcifications, hydrocephalus, polymicrogyria, cortical atrophy, and skull asymmetry (2). Skin abnormalities include alopecia, nevus psiloliparus, and masses of adipose subcutaneous tissue in the frontal-temporal and zygomatic area. Ocular abnormalities include choristoma, epibulbar or limbic dermoids, ocular or palpebral colobomas, aniridia, microphthalmia, calcifications of the globe, and irregular eyebrows shape (2).

Up to 10% of pediatric patients developing a cerebral tumor present an underlying genetic syndrome which contributes to an increased neoplastic risk. This has been observed especially in Neurofibromatosis, Tuberous Sclerosis, Gorlin syndrome, and Turcot syndrome. It is, therefore, possible to hypothesize that Haberland syndrome, similarly to most of the neurocutaneous syndromes, is associated with a predisposition to cancer development. Besides typical benign non-progressive intracranial or spinal lipomas, other neoplasms have been documented including ossificant fibromas, odontomas, and osteomas (6–9). Brain tumors are extremely rare in Haberland syndrome; to our knowledge, only four cases have been reported to date. The first description is attributed to Brassesco et al., who provided the report about a 3-year-old male patient with a pilocytic astrocytoma located at the suprasellar region and extending throughout the hypothalamus and third ventricle (10). Following this, the case of a 7-year-old female with a papillary glioneuronal tumor located at the floor of the third ventricle was published by Phi et al. (11). Subsequently, Valera et al. described a 3-year-old female with a pilocytic astrocytoma adjacent to the left internal capsule and hypothalamus. Moreover, they raised the idea that Haberland syndrome, similarly to other cancer-prone genetic disorders, might be associated with an increased risk of developing low-grade gliomas (12). The case of a 32-year-old woman with a glioblastoma of the right temporal lobe was reported by Fukaya et al. (13).

We report on the first case of pediatric glioblastoma associated with Haberland syndrome. The previously reported pediatric cases included benign brain tumors.

To our knowledge, this is the fifth case of brain tumor associated with encephalocraniocutaneous lipomatosis and the second case of glioblastoma associated with this syndrome.

The hypothesis that Haberland syndrome is associated with an increased risk of tumor development is intriguing, although the rarity of the condition is nowadays preventing us from drawing definitive conclusions about this potential link between the two entities. Further studies are needed to establish the real relationship between encephalocraniocutaneous lipomatosis and the risk of brain tumors.

## Data Availability Statement

The original contributions presented in the study are included in the article/supplementary material, further inquiries can be directed to the corresponding author/s.

## Ethics Statement

Authors obtained written informed consent from the patient's parents for the publication. Written informed consent was obtained from the minor(s)' legal guardian/next of kin for the publication of any potentially identifiable images or data included in this article.

## Author Contributions

SF, IS, MG, and SG contributed to patient's care and wrote the first draft of the manuscript. CL contributed to the final version of the manuscript. All the authors contributed to manuscript revision, read, and approved the submitted version.

## Conflict of Interest

The authors declare that the research was conducted in the absence of any commercial or financial relationships that could be construed as a potential conflict of interest.
